# Implementation of the Robson classification for caesarean sections in Israel: a 10-year cross-sectional study

**DOI:** 10.1186/s13584-025-00726-z

**Published:** 2025-11-04

**Authors:** Noa Shtainmetz, Riki Tesler, Nachman Ash, Liat Korn

**Affiliations:** Department of Health Systems Management, School of Health Sciences, Ariel University, Ariel, Israel

**Keywords:** Caesarean section, Caesarean delivery, Healthcare, Robson classification system

## Abstract

**Background:**

In recent decades, caesarean section (CS) rates have dramatically increased; the reasons for this trend are multifactorial and not fully understood. This continuing trend has raised public health concerns regarding higher maternal and perinatal risks, high costs, healthcare efficiency, and inequality of services. We aimed to characterize and evaluate Israel’s CS rate by applying the Robson classification system.

**Methods:**

This was a national retrospective cross-sectional study. Data from 1,061,786 live births were collected from electronic medical records of women admitted for delivery across all Israeli hospitals between 2014 and 2023. The Robson classification system (also known as the Robson ten group classification system; RTGCS), has been used to monitor, assess, and compare CS rates. Data analysis followed WHO’s RTGCS manual guidelines. Each birth was classified into one of the Robson groups to assess group size, the CS rate within each group, and the contribution to the overall CS rate.

**Results:**

We found an increasing trend in CS rates, with 19.0% marking the highest rate recorded over the last decade. Group 3 + 4 (multiparous, singleton, cephalic, term pregnancy without previous CS) and 1 (nulliparous, singleton, cephalic, term pregnancy in spontaneous labor) were the most represented (56.1% and 20.7%, respectively). The major contributors to CS included Groups 1, 3 + 4, 5 (multiparous, singleton, cephalic, term pregnancy with a previous CS), 8 (all multiple pregnancies), and 10 (singleton, cephalic, pre-term pregnancy).

**Conclusions:**

Using a population-based dataset that spanned ten years, this study identified subgroups in need of targeted interventions and offered insight into CS rate dynamics. The study underscores the RTGCS’s potential to optimize maternity outcomes, shape policy, and inform healthcare practices, making a meaningful contribution to the field. The findings highlight the importance of integrating RTGCS into routine data collection and improving obstetric data quality.

## Background

Caesarean sections (CS) are widely recognized childbirth procedures that have been in use since the 1500 s and have become a cornerstone of modern obstetrics and gynecology practice [1]. To date, CSs serve as vital and life-saving procedures in reducing maternal and neonatal morbidity and serve as a maternal health services quality indicator. CSs are often performed as essential procedures for both mothers and newborns in cases of complications, such as antepartum hemorrhage, fetal distress, abnormal fetal positioning, or hypertensive disorders [2].

Despite their proven advantages, CSs can increase medical risks for mothers and newborns, especially when performed without a medical indication [3–5]. Compared to vaginal birth (VB), CS delivery is associated with a five-fold or even greater increase in postpartum mortality [6]. Other notable previously reported outcomes that are associated with the underuse or overuse of CS include short- and long-term adverse perinatal maternal morbidity [4, 7], including infections, hemorrhaging, anesthesia-related complications, adhesions, lacerations, extended recovery times, and the risk of additional surgeries [8]. Previous studies have also reported that following CS, there are increased negative emotional and psycho-social outcomes regarding the mother-baby relationship and attachment, a higher risk of postpartum depression, less skin-to-skin contact, and unsuccessful breastfeeding as compared to VB [9–11].

Additionally, the hormonal, physical, and bacterial exposures of newborns born by CS are different than those born by VB; those born by CS often experience difficulties establishing the gut microbiota and are at a higher risk of developing allergies, atopic dermatitis, breathing problems, asthma, childhood onset type 1 diabetes, obesity in adulthood, and receiving a low Appearance, Pulse, Grimace, Activity, Respiration (Apgar) score [4, 10, 12].

Furthermore, CSs are associated with higher costs than VBs, which can inflict financial strain on a healthcare system, as hospital stays are usually longer and recovery can be more complicated [7, 10, 13, 14]. According to the Israeli Ministry of Health and the Organization for Economic Co-operation and Development (OECD), the cost of CS delivery is estimated to be more than twice that of VB [15]. In light of this, CS characterization, improvement, and optimization are currently a global health priority [16, 17].

CS has increased rapidly in developed and developing countries in recent decades without fully understanding its underlying causes. Yet, the global average is now 21.1%, with a consistent upward trend. Estimates indicate that by 2030, 28.5% of women worldwide will have given birth by CS [16–20]. This trend has also been apparent in Israel [21]. According to the Israeli Ministry of Health [15] and the Israeli Society for Maternal and Fetal Medicine [22], the CS rate has risen by 80% since 1999; roughly one out of every five women in Israel who have given birth has done so by CS [23]. Approximately 6,000 CSs are undertaken in Israel annually with no medical indication, translating to about 3.6 million dollars spent unnecessarily on healthcare costs (i.e., based on the marginal resources directly associated with CS procedures) [24].

According to the World Health Organization (WHO) in 1985, the CS rate should ideally not be higher than 10%−15% of all deliveries [25]; in 2015, the organization put out a statement that CS rates above 10% are not associated with a decrease in maternal or newborn mortality rates [26]. This statement has been supported by findings in recent studies [27–29]. Nevertheless, CS should be provided based on necessity, rather than aiming to achieve a specific rate [30].

The lack of a standardized, internationally accepted classification system to monitor and compare CS rates in a consistent and action-oriented manner is one of the barriers to a deeper understanding of this trend [20, 31]. It is crucial to be able to monitor and compare CS rates across countries; rather than setting a target for CS rate reduction based on arbitrary goals, there is a need for an evidence-based approach [32, 33].

**Fig. 1:**
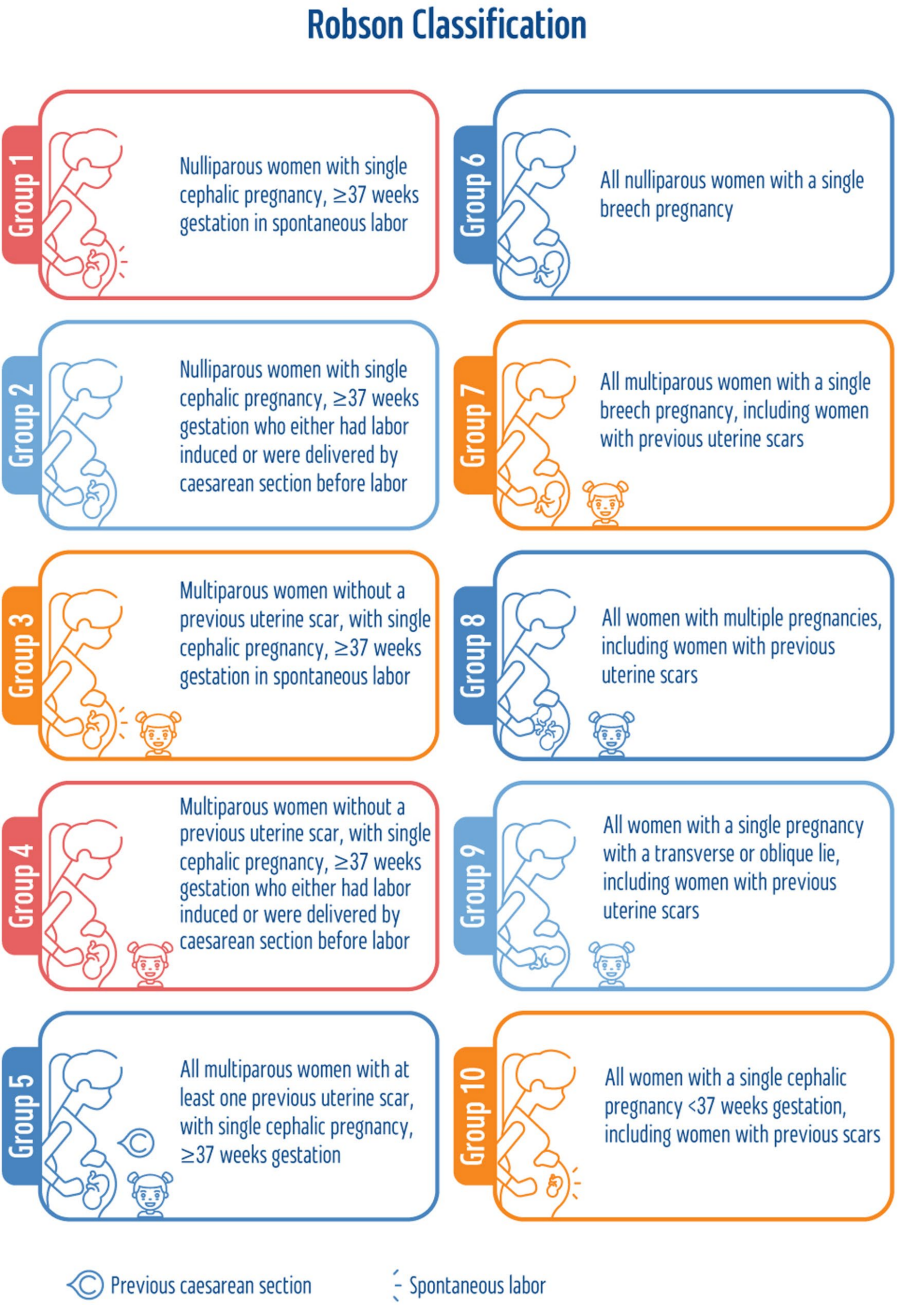
The ten group classification system. (Source: the authors)

Since 2015, the WHO has recommended the adoption and application of the Robson Classification System, also known as the “Robson Ten Group Classification System” (RTGCS), as the most valid method to meet local and international perinatal and CS monitoring needs, as well as assess and compare CS rates between and within healthcare facilities [34, 35]. RTGCS classifies all women admitted for delivery into one of ten mutually exclusive and inclusive groups based on predefined obstetric indicators [36]: parity and previous CS, onset of labor, number of fetuses, gestational age, and fetal presentation. These simple indicators are routinely collected during hospital admission for delivery [37]. Figure 1 describes the clinical characteristics of each group.

The RTGCS serves as an overall prospective structure in which it is possible to characterize, monitor, evaluate, and compare CS rates within and among hospitals [21, 38]. This classification has been recognized in a wide variety of health systems and among policymakers and health experts worldwide, as it offers an opportunity to effectively monitor and compare CS rates and birth outcomes on a large scale [39–42]. Although the RTGCS is widely considered an applicable system, Israel has not yet implemented it and currently lacks an accepted method for characterizing and monitoring CS rates.

We aimed to characterize CS use in Israel in a 10-year cross-sectional study, through the first-time application and implementation of the RTGCS. Our objective was to estimate the CS rate and conduct an analysis based on the five indicators (obstetrical history – parity & previous CS, onset of labor, number of fetuses, gestational age, and fetal presentation). The initial assumption is that a CS rate is considered appropriate and optimal only if there is sufficient data to justify and explain it [17]. Hence, we aimed to evaluate whether implementing the RTGCS in routine maternity data collection across all hospitals in Israel for the first time will enable meaningful, reliable, and actionable monitoring of CS rates to ensure optimal usage.

## Methods

### Study design and setting

A national retrospective cross-sectional study was conducted. All hospitals with labor and delivery units in Israel were included (*N* = 26, with 13 in the central district, 9 in the northern district, and 4 in the southern district). Data were obtained from the electronic medical records of women admitted for delivery and from the Ministry of Health’s TIMNA Big Data platform (Israel’s National Health Research). We included women who were admitted for delivery between January 1, 2014, and December 31, 2023. We excluded those with missing data on key Robson indicators (‘core variables’) in the medical record. All stillbirths were also excluded from the study.

## Variables

### Independent variables

Obstetrical indicators included: (1) Obstetrical history (Parity; nullipara; multipara; previous CS); (2) Onset of labor (spontaneous; induction [initiation of uterine contractions by medical or surgical means before the onset of natural labor], pre-labor CS); (3) Number of fetuses (singleton, multiple); (4) Gestational age (pre-term [delivery at < 37 weeks gestation); On-Term (delivery at *≥* 37 weeks gestation]); and (5) Fetal presentation (cephalic, breech, oblique lie).

Maternal socio-demographic variables (age, ethnic group, place of birth, and marital status).

Maternal, newborn, and labor characteristics (mode of delivery, Apgar score 1 min, Apgar score 5 min, newborn’s birth weight [g]).

Number of live births each year (1-1-2014–31-12-2023).

## Dependent variables

CS rate (from all live births).

## Procedure, data extraction, and analysis

**Fig. 2:**
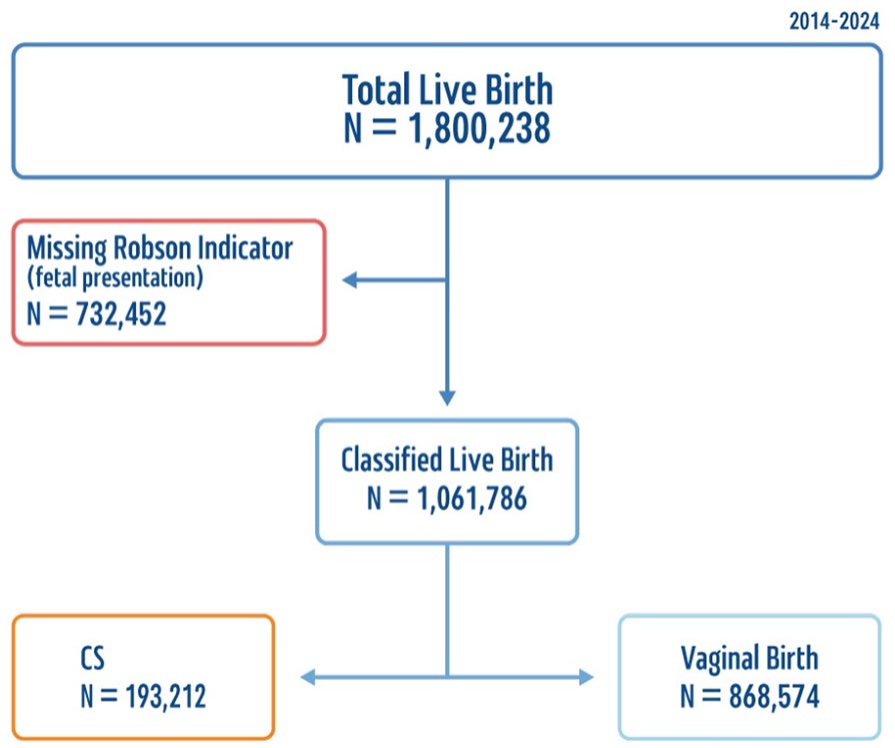
Flowchart for missing values

Data were retrieved and analyzed according to the recommendations of the RTGCS manual and synthesized according to the standardized reporting tables provided by the manual [43]. According to the WHO methodology, the analysis should follow several key steps. First, births that could not be classified due to missing data in at least one of the core variables were excluded (Fig. 2).

Second, the overall CS rate from all live births was calculated, including trends over the years. Third, each birth was classified into one of the Robson groups, using the five core variables (obstetric history – parity and previous CS, onset of labor, number of fetuses, gestational age, and fetal presentation). The groups were translated into Hebrew for the first time as part of the current study. CS rates were calculated as a percentage relative to the obstetric population within each Robson group. The absolute contribution represented the proportion of CS cases relative to the total obstetric population, while the relative contribution reflected the proportion of CS within each Robson group compared to the total number of CS cases.

In addition, as recommended in the WHO manual [43], relevant additional information provided by the local data collection system was used as complementary information to allow for an in-depth interpretation of CS practices. Specifically, the background and socio-demographic variables of the mother were sampled and analyzed. For each step, findings were compared with the suggested source of interpretation in the WHO manual.

The births included in this study were characterized by four maternal backgrounds and socio-demographic variables (age, categorized into the following groups: >20, 20–29, 30–39, 40–49, and *≥* 50 years [44]; ethnic group [Jewish, Muslim Arab, Druze, Bedouin and other]; marital status [married, single, divorced, widowed and other]; place of birth [Israel, Ethiopia, Russia and Ukraine, America, Europe and other]), maternal, newborn, and labor characteristics (obstetric history, mode of delivery, number of fetuses, gestational age, fetal presentation, number of prior birth, birth weight [categorized as high, normal, or low: over 4000 g (g), 2500–4000 g, and under 2500 g, respectively] [45], and Apgar scores [high: *≥*7–10, low: <7, measured at 1 min and 5 min after birth]). The one- and five-minute Apgar scores offer a repeatable method for assessing a newborn’s health status and vital signs immediately after birth. This scoring system evaluates five key criteria: skin color, pulse rate, reflex response, muscle tone, and respiratory effort on a scale from zero to ten [46]. All variables were analyzed using descriptive statistics, including frequencies and percentages [47].

The dataset was constructed from three independent national electronic databases: Maternal and Child Health Clinics (“Tipat Chalav”), Birth Registry, and Hospitalization Registry. As all records were linked using the encrypted maternal and newborn ID numbers (consistent across all datasets), we were able to perform validation steps: Multiple cross-validations across the different registries. These cross-checks ensured that key variables (e.g., parity, fetal presentation, delivery mode) were consistent and yielded identical results across all sources.

Data analysis was conducted in the TIMNA research environment, with de-identified data provided by encryption specialists who managed access to the coding key. The authors analyzed the data using a virtual personal work environment via a remote connection. Statistical processing was performed using the Dataiku Statistical Environment. The level of significance was set at α = 0.05. The analysis of RTGCS data relied on simple arithmetic counts and proportions described in the WHO Implementation Manual [43].

### Ethics

The study was approved by Ariel University’s Ethics Committee (approval no. AU-HEA-LK-2023111) and Israeli Ministry of Health’s Supreme Committee for Human Experiments and the Information Committee (Approval No. 862, 7a). The study did not require any direct contact with patients and it could not cause any harm to them. The data organization and analysis strictly followed ethical guidelines aligned with the Helsinki Declaration for human research. All data sources were fully de-identified, ensuring compliance with k-anonymity standards. The data were securely stored following regulations for medical information collection and retention.

## Results

### Live birth, the overall CS rate, and trends over the years

As presented in Table 1, a total of 1,061,786 live births were included, with annual counts ranging from approximately 106,000 to 117,000. Over the study period, 193,212 CS deliveries were performed, reflecting an overall CS rate averaging 18.2%. Notably, this rate was also consistent among the 1,800,238 live births that could not be classified due to missing one of the Robson core variables.

**Fig. 3:**
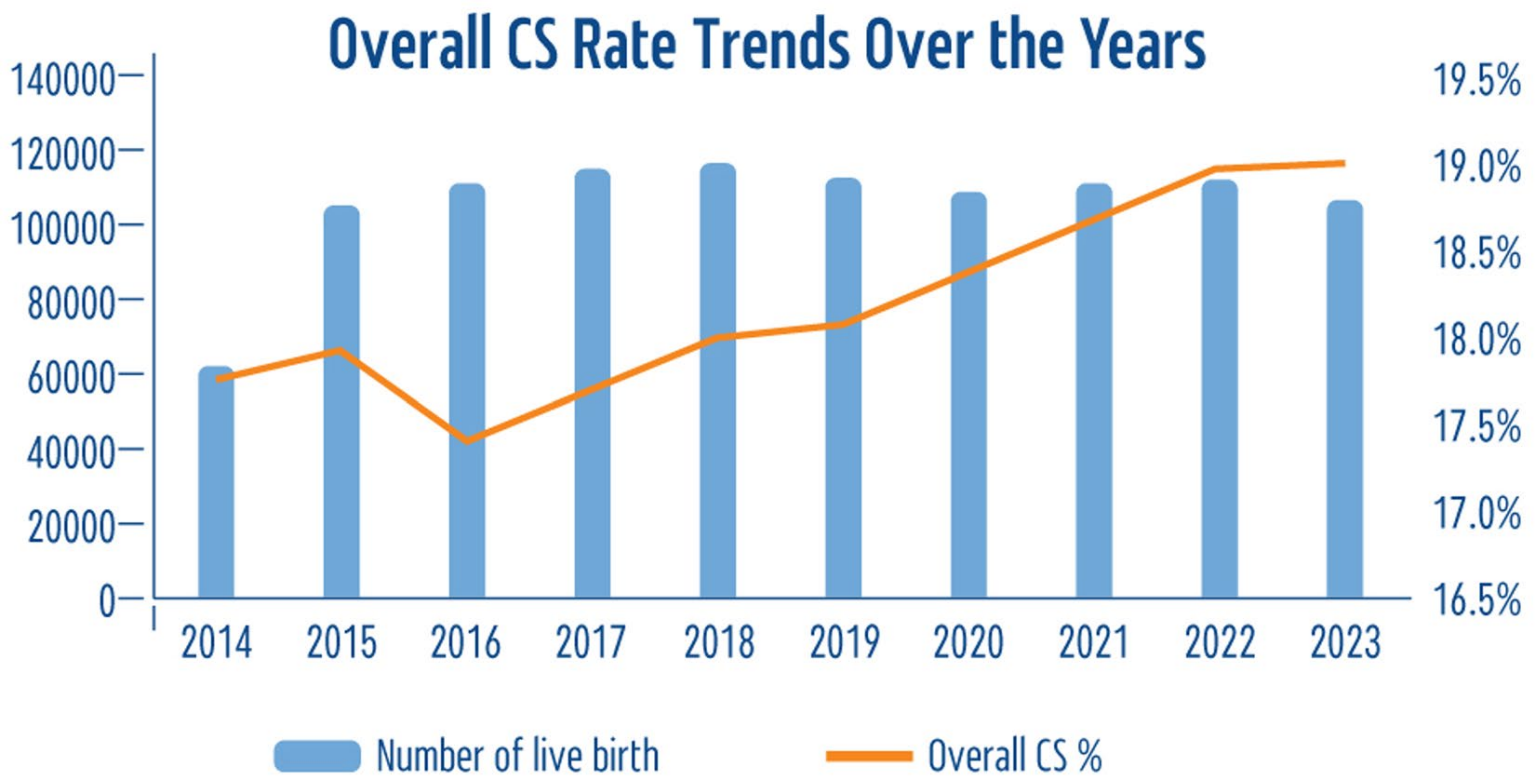
Overall caesarean section rate trends over the years (of all Live Births)

We saw a clear increasing trend in CS rates, from 17.8% in 2014 to 19.0% in 2023, with a gradual, consistent rise each year except for a minor decline in 2016 that was subsequently offset in 2017 (Fig. 3). It is worth noting that while the Israeli Ministry of Health’s databases show slight data gaps in 2014, the CS rate that year remained consistent with other years. A key finding from Table 1 highlights that the CS rate in Israel reached a peak of 19.0% for 2022–2023, marking it the highest rate recorded over the last decade.

**Table 1:** Live birth and overall caesarean section rate 2014–2023 (*N* = 1,061,786)

Year	Number of live births	Number of Caesarean sections	Cesarean sections’ rate (%)
**2014**	62,327	11,074	17.8%
**2015**	105,505	18,902	17.9%
**2016**	110,902	19,310	17.4%
**2017**	114,976	20,360	17.7%
**2018**	116,689	20,994	18.0%
**2019**	113,068	20,441	18.1%
**2020**	108,769	19,969	18.4%
**2021**	110,925	20,660	18.6%
**2022**	111,667	21,177	19.0%
**2023**	106,958	20,325	19.0%
**Total**	1,061,786	193,212	18.2%

### Maternal, newborn, and labor characteristics

During the study period, most live births occurred among women aged 30–39 (49.5%), followed by those aged 20–29 (44.1%; Table 2). Births among women aged 40–49 accounted for 5%, while births among women under 20 (1.7%) and over 50 (< 0.1%) were less common. Most live births were of married women (88.1%), with fewer single (3.5%), divorced (1.0%), and widowed women (0.1%). Marital status was missing or listed as “other” for 7.3% of the study population. Most mothers were Jewish (62.8%), followed by Muslim Arabs (22.1%), with smaller groups including Bedouins (2.5%) and Druze (1.9%). Ethnic groups were missing for 10.8% of cases. Most women were Israeli-born (83.9%), with the largest foreign-born groups from Russia and Ukraine (5.5%), and fewer from Ethiopia, Europe, and America. Nearly a third (30.1%) of mothers were nulliparous, while 69.9% were multiparous.

The majority of births (75.6%) were on-term (delivery at ≥ 37 weeks gestation), while 5.7% were pre-term (delivery at < 37 weeks gestation). The vast majority of the population had single pregnancies, comprising 96.0%, with only 4.0% having multiple pregnancies. Most were in a cephalic position (94.8%), followed by breech presentations (3.7%). As for birth weight, the majority (87.7%) fell within 2500–4000 g, indicating a normal weight range. Low birth weight (< 2500 g) was observed in 7.3% of births, and 5.0% had a birth weight over 4000 g.

The mode of delivery was predominantly by vaginal birth (VB; 76.5%). Operative vaginal births (O-VB) constituted 5.3%, while CSs accounted for 18.2%. Apgar scores at 1 and 5 min indicated generally high newborn health scores. At 1 min, 95.9% of newborns had high Apgar scores (7–10), while only 2.3% scored below 7. At 5 min, 97.1% had high Apgar scores (7–10), with just 0.4% scoring below 7.

**Table 2:** Maternal, newborn and labor characteristics, 2014–2023 (Live Births, *N* = 1,061,786)

Characteristics		Frequency (*n*)	Percentage (%)
**Maternal age**,** in years**	< 20 20–29 30–39 40–49 > 50	17,918 525,911 467,919 49,513 525	1.7% 44.1% 49.5% 4.7% < 0.1%
**Marital status**	Married Divorced Single Widowed Other	941,521 11,001 37,623 568 78,131	88.1% 1.0% 3.5% 0.1% 7.3%
**Ethnic group**	Jewish Muslim Arab Bedouin Druze Other	661,328 233,320 25,849 19,657 113,271	62.8% 22.1% 2.5% 1.9% 10.8%
**Maternal ** place of birth*	Israel Russia and Ukraine Ethiopia Europe America Other	896,646 58,816 18,396 15,350 16,771 56,046	83.9% 5.5% 1.7% 1.4% 1.6% 5.2%
Gestational age (weeks) at delivery**	Pre-term; <37 weeks	60,436 802,702	5.7% 75.6%
	On-term; >37 weeks		
**Type of pregnancy**	Single	1,018,982 42,803	96.0% 4.0%
	Multiple		
**Parity**	Nulliparous	320,022 741,764	30.1% 69.9%
	Multiparous		
**Newborn position**	Cephalic Breech Oblique lie	1,006,166 39,338 16,282	94.8% 3.7% 1.5%
**Mode of delivery**	Vaginal birth (VB)	812,034 56,539 193,212	76.5% 5.3% 18.2%
	Operative vaginal birth (O-VB)		
	Cesarean section (CS)		
**Apgar score at ** 1 min***	High (*≥* 7–10)	1,018,690 24,091	95.9% 2.3%
	Low (< 7)		
**Apgar score at** 5 min****	High (*≥* 7–10)	1,031,221 4,148	97.1% 0.4%
	Low (< 7)		
**Newborn’s birth weight**,** in grams**	< 2500	77,156 931,366 53,264	7.3% 87.7% 5.0%
	2500–4000		
	> 4000		

*Maternal place of birth: less than 1.0%: Asia (4824, 0.5%), Africa (1,886, 0.2%), Australia (409, 0%)

**Gestational age at delivery: 18.7% had missing values

***Apgar score at 1 min: 1.8% had missing values

****Apgar score at 5 min: 2.5% had missing values

### Robson classification analysis

Table 3 presents the results of the RTGCS analysis. Because data on induced multiparous women were not separated from all multiparous women, Groups 3 and 4 (multiparous, singleton, cephalic, term pregnancy without previous CS) were combined into Group 3 + 4.

Group 1 (nulliparous singleton, cephalic, term pregnancy in spontaneous labor) comprised 20.7% of the maternity population, with a CS rate of 12.5%. This group contributed an absolute 2.6% to the overall CS rate, accounting for 14.2% of the relative group contribution. Group 2 (nulliparous singleton, cephalic, term pregnancy, induced or CS before labor) comprised 4.1% of the population with a 16.9% CS rate, contributing 0.7% to the overall CS rate and 3.8% in its relative contribution.

Group 3 + 4 (multiparous, singleton, cephalic, term pregnancy without previous CS) represented the largest portion of the population at 56.1%, and had a low CS rate of 4.4%, resulting in a 2.5% absolute contribution and a 13.5% relative contribution. Group 5 (multiparous, singleton, cephalic, term pregnancy with a previous CS) comprised 5.1% of the population and had the highest CS rate among all groups at 63.4%. This group contributed the largest absolute proportion (3.2%) and relative contribution (17.8%) to the overall CS rate.

Groups 6 and 7 (all breech positions) had high CS rates, with Group 6 at 93.9% and Group 7 at 87.9%. These groups contributed 1.4% and 1.9% to the overall CS rate, respectively, accounting for 7.7% and 10.7% in relative group contributions. Group 8 (all multiple pregnancies) comprised 4.0% of the maternity population, with a CS rate of 61.4%, contributing 2.5% to the absolute CS rate and 13.6% in relative contribution. Group 9 (all abnormal fetal presentations) represented 1.5% of the population with a CS rate of 62.1%, contributing 1.0% to the absolute CS rate and 5.2% in relative contribution. Group 10 (singleton, cephalic, pre-term pregnancy) represented 4.7% of the population, with a CS rate of 52.1%, contributing 2.5% to the absolute CS rate and 13.5% in relative contribution.

The RTGCS analysis provides valuable insight into CS trends within the maternity population in Israel. **In conclusion**, Group 3 + 4 and Group 1 were the most represented groups (56.1% and 20.7%, respectively). Group 5 was the third most represented group (5.1%). The major contributors to CS were as follows: Group 5 (17.8%); Group 1 (14.2%); Group 8 (13.6%); Group 3 + 4 (13.5%); Group 10 (13.5%).

**Table 3:** The robson classification report table (*N* = 1,061,786)

Robson Group	Number of CS/number of deliveries in group	Group size	Group CS rate	Absolute group contribution to overall CS rate	Relative group contribution to overall CS rate
1	27,351/219,277	20.7%	12.5%	2.6%	14.2%
2	7,413/43,793	4.1%	16.9%	0.7%	3.8%
3 + 4	26,136/596,116	56.1%	4.4%	2.5%	13.5%
5	34,309/54,115	5.1%	63.4%	3.2%	17.8%
6	14,849/15,817	1.5%	93.9%	1.4%	7.7%
7	20,672/23,521	2.2%	87.9%	1.9%	10.7%
8	26,275/42,804	4.0%	61.4%	2.5%	13.6%
9	10,104/16,282	1.5%	62.1%	1.0%	5.2%
10	26,103/50,061	4.7%	52.1%	2.5%	13.5%
Total	193,212/1,061,786	100%	18.2%	18.2%	100.0%

Absolute contribution (%) = n of CS in the group/total N of deliveries in the hospital x 100

Relative contribution (%) = n of CS in the group/total N of CS in the hospital x 100

Absolute contribution (%) = n of CS in the group/total N of deliveries in the hospital x 100.

Relative contribution (%) = n of CS in the group/total N of CS in the hospital x 100.

**Fig. 4:**
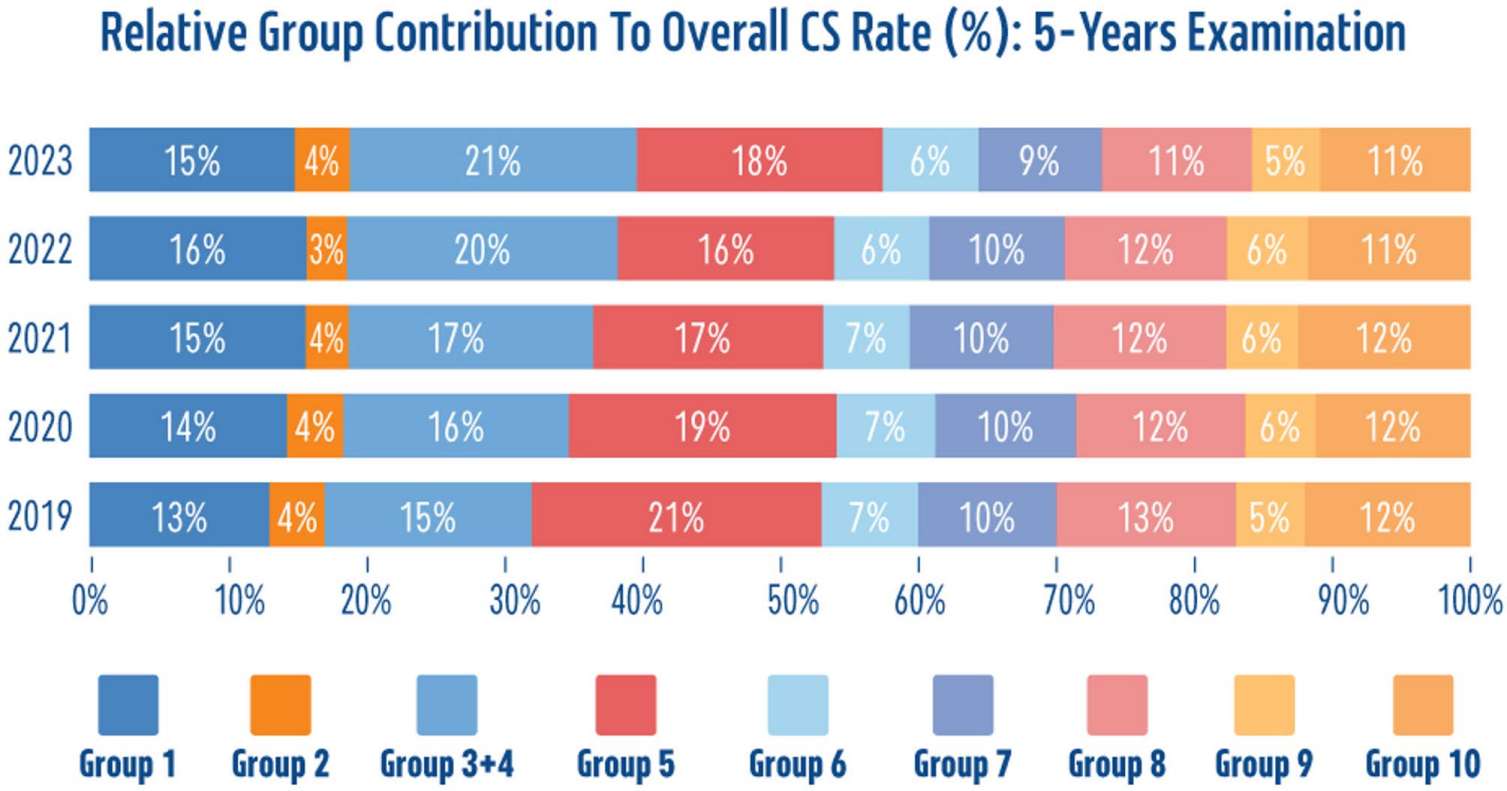
Relative group contribution to overall CS rate: 5-year examination (*n* = 104,332)

An in-depth analysis of the relative contribution of each group to the overall CS rate over the past five years (2019–2023) revealed several important trends in CS rates across different RTGCS groups (Fig. 4).

Group 1 consistently contributed substantially to the overall CS rate, with a gradual increase observed from 13% in 2019 to 16% in 2022, with a slight decrease to 15% in 2023. This trend indicates a rising demand for CSs among first-time mothers in spontaneous labor. The contribution from Group 2 remained low and stable, between 3 and 4%, suggesting that induction or pre-labor CS among first-time mothers was a less influential factor in the overall CS rate.

Group 3 + 4 consistently represented the largest contribution to the CS rate, ranging from 15% to 21%. The group’s substantial contribution highlights a high incidence of CSs among multiparous women without a previous CS history, with a steady increase from 2019 to 2023. Group 5 maintained a significant and stable contribution to the overall CS rate, ranging from 16% to 21%, with a slight decline over the years. The consistent impact of this group emphasizes the influence of a prior CS on the likelihood of repeat CS deliveries, with probably more vaginal births after CS (VBAC) being performed in recent years.

Groups 6 and 7 contributed to approximately 6% and 10%, respectively, underscoring breech presentation as a significant factor in determining CS rates, particularly among multiparous women. Group 8 has a steady contribution of approximately 11–13% annually, which reflects the higher probability of CS in cases involving multiple pregnancies. Group 9’s low but stable 5%−6% contribution across the years suggests that transverse or oblique fetal positions were an infrequent yet consistent reason for CS. Group 10 showed a steady contribution of about 11–12% each year, indicating that preterm deliveries played an influential role in the overall CS rate.

## Discussion

A primary goal of maternity care is to provide safe outcomes for mothers and newborns during childbirth. The worldwide CS rate has increased dramatically in recent decades and the reasons for this trend are not fully understood. The continuing rise in CS rates is a public health concern due to higher maternal and perinatal risks, high costs, the need for healthcare efficiency, and inequality of access to services [17, 19, 48]. The current study aimed to characterize and optimize CS use in Israel by applying and implementing the RTGCS.

We found that a total of 193,212 CS deliveries were performed between 2014 and 2023, reflecting an overall CS rate averaging 18.2%. Examining trends over time revealed a clear rising trend in CS rates, peaking at 19.0%.

The RTGCS’s first-time implementation in Israel is at the heart of the current study. In the current research, 732,452 deliveries (40.6%) could not be classified in any of the Robson groups. Upon reviewing these specific records, it was seen that the missing information was fetal presentation.

However, to assess potential bias arising from missing data, we compared the available demographic and clinical characteristics of births classified within the RTGCS to those that could not be classified due to missing fetal presentation. The distribution of maternal age, parity, plurality, and mode of delivery was similar between the two groups, with no statistically significant differences in overall CS rates. Minor variations were observed in geographic distribution and hospital type, reflecting differences in data completeness across facilities. These findings suggest that the excluded unclassified births are broadly representative of the national birthing population, and their omission from the Robson classification analysis is unlikely to introduce substantial selection bias. Nevertheless, we acknowledge that missingness on a core Robson variable limits the ability to fully assess group-specific CS rates. As fetal presentation is a relatively simple variable to document, it is recommended that medical teams ensure its inclusion in medical records to reduce the number of unclassifiable cases and improve data quality in the future.

We interpreted our results following the recommendations provided by WHO in 2017. The classification revealed that Groups 1, 3 + 4, and 5 were the most represented. The major contributors to CS were as follows: Group 5, which emphasizes the influence of a prior CS on the likelihood of repeat CS deliveries; Group 1, which indicates a rising demand for CS among first-time mothers in spontaneous labor; Group 8, which reflects the higher probability of CS in cases involving multiple pregnancies; Group 3 + 4, which highlights a high incidence of CS among multiparous women without a previous CS history; and Group 10, which indicates that pre-term deliveries play a significant role in the overall CS rate.

The five-year analysis (2019–2023) revealed critical insights into the factors influencing these rates, highlighting consistent patterns in certain population subgroups and providing valuable guidance for healthcare policy and obstetric practice. Overall, the data illustrate that multiparity (especially with previous CS history), multiple pregnancies, and pre-term deliveries are key drivers in Israel’s CS rates, alongside a worrying trend indicating a rising demand for CS among first-time mothers in spontaneous labor; from 13% in 2019 (2,766 CSs) to 15% in 2023 (3,204 CSs).

It is crucial to focus on interventions in group 1, as this group comprises women experiencing their first birth. Group 1 is considered in the literature as an obstetric low-risk group; which allegedly has no medical justification for CS. Reducing the number of CS in nulliparous women would subsequently decrease the likelihood of repeat CSs in the future and might prevent unnecessary CS. An increase in group 1 has also been documented in recent studies from other countries [49, 50].

Another notable contribution to the CS rate relates to the prevalence of group 5 (multiparity with previous CS), even though there was a relatively slight decline over the years, which may suggest that perhaps more VBACs are being performed. A comprehensive review of 21 studies conducted in different countries, such as Canada, Australia, and Brazil revealed that group 5 was the most vulnerable group and the primary contributor to overall CS delivery rates. Together, the prominence of group 1 and group 5 found in the present study underlines a strong “cascade” effect – a secondary effect - where an initial CS significantly increases the likelihood of subsequent CS deliveries, reinforcing the need to evaluate first-time CS carefully. This is also relevant to Group 3 + 4, which highlights a high incidence of CS among multiparous women without a previous CS history. These groups should prioritize the following specific goals: avoiding the first CS, whether during labor or before it begins; and challenging the belief that “once a cesarean, always a cesarean” [51]. Refraining from scheduling repeat or medically unnecessary CS could be an effective strategy to reduce CS in these hospitals.

Regarding group 8, which reflects the higher probability of CS in multiple pregnancies, due to the difficulties inherent to twin births and the need for skilled professionals trained to deliver twins (or more) [51], it would be motivating for hospitals to invest in the training of a specific team for this type of birth. Group 10, which indicates a higher probability of CS in pre-term deliveries, is frequently mentioned in the literature as a key factor contributing to the high CS rates in many delivery units, particularly in tertiary referral centers, although this may not always be the case. It represents the group of women where there is likely the most consistent management approach by obstetricians to opt for CS, due to the limited alternatives. The overall contribution of this group to the CS rate is therefore typically more influenced by its size rather than the CS rate within the group itself. It is important to evaluate this group based not only on previous obstetric history but also on the progression of the current pregnancy. According to Robson [36], safely reducing the CS rate in this group would be challenging, though the possibility depends on the initial rate. The more standardized data collected from various units, and over time within the same unit, the greater the potential for meaningful comparison and improvement.

International experience demonstrates that RTGCS-guided quality improvement initiatives can lead to measurable reductions in CS rates when integrated with targeted, context-specific interventions. For example, in Brazil, the national “Project Appropriate Birth” (PPA) implemented a multifaceted strategy that included organizational restructuring, women’s education and engagement, continuous monitoring of Robson groups, and adoption of evidence-based intrapartum care models. Over the course of the program, overall CS rates declined, with a reduction in low-risk Groups 1–4 [40]. Similarly, a quality improvement program targeted Robson Groups 1 and 3 through midwife–obstetrician collaborative care, appointment of a dedicated medical coordinator, daily supervisory rounds, updated labor protocols, and team-based training. This approach achieved a relative reduction of in Group 1 and Group 3 CS rates, alongside an overall decrease among women cared for by the in-house team [52]. These examples highlight that the RTGCS is not only a monitoring tool but also a practical framework for driving targeted, data-driven interventions, and that coupling its implementation with regular feedback, clinical governance, and staff training can yield sustained improvements in obstetric care.

Empirical evidence has shown that the RTGCS can easily be adapted for local use. The size and CS rate of each of the ten groups can provide useful information about care quality in a particular region or setting and be used to compare an intervention’s impact. Therefore, the RTGCS data, based on a perinatal classification system, can provide valuable insight regarding not only the CS rate but also obstetric populations and outcomes [39–41, 53, 54]. For the first time, it is possible to compare and characterize Israeli CS rates and usage patterns, as well as the great importance of its implementation in health services. The RTGCS provides an initial structure for analyzing multiple epidemiological variables, processes, and perinatal outcomes. In this context, it may serve as a promising starting point to accommodate changing landscapes and to align with evolving medical and societal dynamics [55, 56].

## Conclusions

This exploratory study offers the first comprehensive analysis of the RTGCS in Israel and the need for adopting it as a standardized tool to optimize labor and delivery practices. The study identifies specific obstetric conditions, such as previous CSs and multiple or pre-term pregnancies as persistent contributors to CS-related decisions. More specifically, our study confirmed that Groups 1, 3 + 4, 5, 8, and 10 were identified as “target groups”. These target groups require more in-depth analysis to identify possible modifiable factors and to apply specific interventions and strategies to reduce the CS rate. Strategies could include providing additional support for VBACs where appropriate, enhanced training for multiple-pregnancy deliveries, and ongoing monitoring to prevent unnecessary first-time CSs. Addressing these factors systematically could contribute to a broader goal of reducing overall CS rates, aligning with evidence-based practices to improve maternal and fetal outcomes.

Furthermore, data collection and interpretation are central to medical treatment and decision-making. We suggest that rather than goal-oriented data collection, a contemporary and more up-to-date research paradigm should be based on big data collection through RTGCS implementation and analysis with advanced tools. Such a study design and focus would likely lead to new insights regarding CS rates and areas in which benchmarking can be improved.

### Practical and clinical implications

The RTGCS, based on routinely collected hospital data, offers valuable insight for advancing maternity health. It enables reliable monitoring of CS rates, supports data-driven policy discussions, and facilitates hospital benchmarking locally and globally [17]. To our knowledge, our study marks the first application of the RTGCS in Israel, providing baseline data for tracking trends and informing comparisons. The findings highlight the importance of integrating RTGCS into routine data collection and improving obstetric data quality.

Using a population-based dataset that spanned ten years, this study identified subgroups in need of targeted interventions and offered insight into CS rate dynamics. The study underscores the RTGCS’s potential to optimize maternity outcomes, shape policy, and inform healthcare practices, making a meaningful contribution to the field. Moreover, Israel’s participation in the global RTGCS database allows for international collaboration, the adoption of effective practices, and enhanced transparency through hospital audits and outcome analyses.

### Limitations and future research

Despite its importance, this research is not without limitations. First, retrospective data collection remains a significant limitation even in large data sets. A follow-up study can improve the data quality and perhaps make it possible to separate Groups 3 and 4. The absence of external validation is another limitation. To minimize it, we validated our results in a separate test data set.

The database, while comprehensive, excluded variables such as prenatal care quality [54], maternal education, cultural influences, access to healthcare, and socio-economic status, which could have influenced the findings [33]. Although the RTGCS identifies contributors to CS rates, it does not explain the underlying causes and key factors such as pre-existing conditions or complications [31].

For example, use of assisted reproductive technologies (ART) has been linked to higher CS rates, even after adjusting for maternal age and pregnancy complications. This is attributed to increased medical risks (e.g., multiple gestations, hypertensive disorders) and a lower threshold for surgical delivery in pregnancies perceived as high-value. Incorporating ART data into the RTGCS framework could help identify whether specific Robson groups, particularly those involving multiple or induced pregnancies, are disproportionately affected, thus guiding targeted interventions [57]. Integration of such variables into the national perinatal registries would substantially enhance the analytical capacity and policy relevance of Robson-based monitoring.

Unstandardized definitions and underreporting in routine data, as highlighted by Zeitlin et al. [51], also pose challenges. Documenting and standardizing core variables, such as labor onset and induction, is critical for refining the RTGCS. Cross-referencing this system with data on gestational diabetes or maternal behaviors could provide deeper insights into CS risk factors.

Finally, an innovative patient-centered approach could further enhance the classification system. For example, patients could contribute personal reasons for choosing CS, such as fear or religious beliefs. Such insight would support tailored interventions, such as addressing fear through childbirth courses or culturally sensitive outreach for specific populations (e.g., tailored for ultra-Orthodox or Arab sub-populations). This approach fosters informed decision-making and personalized, respectful care while leveraging the full potential of the RTGCS.

## Data Availability

No datasets were generated or analysed during the current study.

## Abbreviations

CS: caesarean section
RTGCS: Robson ten group classification system
VB: vaginal birth
OECD: Organization for Economic Co-operation and Development
WHO: World Health Organization
Apgar: Appearance, Pulse, Grimace, Activity, Respiration
VBAC: vaginal births after CS
